# The prevalence of frailty among older adults with maintenance hemodialysis: a systematic

**DOI:** 10.1186/s12882-024-03921-3

**Published:** 2025-01-07

**Authors:** Juanjuan Li, Wenyi Xiao, Lijuan Wang, Miao Zhang, Yurong Ge

**Affiliations:** 1 College of Nursing, Ningxia Medical University, Yinchuan, Ningxia 750004 China; 2 Department of Medical Education, People’s Hospital of Ningxia Hui Autonomous Region, Yinchuan, Ningxia 750002 China

**Keywords:** Maintenance hemodialysis, Older adults, Frailty, Meta-analysis

## Abstract

**Background:**

To evaluate the epidemiological data on the prevalence of frailty and prefrailty in individuals aged 60 years or older on MHD patients.

**Methods:**

PubMed, Web of Science, Embase, CNKI, WanFang, CBM, and VIP were searched from inception to February 2023 using combinations of subject words and free words. The methodological quality of all the selected studies was assessed using the Joanna Briggs Institute Critical Appraisal of Epidemiological Studies Checklist and Newcastle‒Ottawa Cohort Quality Assessment Scale. Random effects meta-analysis was used to pool estimates from different studies. Subgroup analysis and meta-regression were performed to explore potential sources of heterogeneity.

**Results:**

Of the 4,190 documents retrieved, 16 observational studies involving 2,446 participants from 8 countries were included in this systematic review. Among older adults receiving MHD, the overall prevalence of frailty and prefrailty was 41% (95% CI = 34–49%) and 37% (95% CI = 26–48%), respectively, with considerable heterogeneity. The pooled prevalence of frailty was greater among individuals aged > 70 years (45%) than among those aged ≤ 70 years (37%). However, subgroup analyses indicated that the confidence intervals for the age group overlap substantially.

**Conclusion:**

Our research showed that the prevalence of frailty and prefrailty in older patients with MHD are high.

**Trial registration:**

The PROSPERO registration number for this study was CRD42023442569.

**Supplementary Information:**

The online version contains supplementary material available at 10.1186/s12882-024-03921-3.

## Introduction

The issue of the world population aging has become increasingly severe in the 21st century. This significant increase in the number of older individuals will result in a notable increase in age-related diseases [1], such as chronic diseases and geriatric conditions. As a result, this will place a significant burden on individuals, families, and the public healthcare system [2]. Frailty is a common and important clinical syndrome among older adults and has emerged as a critical public health concern. Geriatric syndrome is characterized by age-related declines in multiple physiological systems, leading to a reduced ability to maintain balance and cope with stress [3]. Consequently, frail individuals are at a higher risk of various health issues, including falls, rehospitalization, and mortality [4]. Unlike age or disability, frailty is considered a predisability condition that indicates the need for additional medical care [5].

The definition of frailty is not universally agreed upon in the academic community. There are multiple tools available for assessing frailty, but it is important to note that their quality may differ [6]. The frailty phenotype and deficit-accumulation frailty index (FI) are commonly used measures for assessing frailty [7]. The phenotype approach defines frailty as the presence of three or more of the following five criteria: muscle weakness, slow walking speed, exhaustion, low physical activity, and unintentional weight loss [8]. Prefrailty is considered an intermediate stage between nonfrailty and frailty and represents a dynamic and noticeable process [9]. The Frailty Index method quantifies frailty by assessing deficits in health, such as symptoms, signs, diseases, and disabilities [10]. A greater number of deficits indicates a greater level of frailty [11]. Despite the various criteria used to define frailty, numerous studies have consistently demonstrate a high prevalence of frailty in older adults [12, 13]. For example, a review of combined results from 21 studies concluded that among more than 60,000 community-dwelling older adults, the average prevalence of frailty was 10.7%, with an additional 41.6% having prefrailty [14]. There is a growing body of evidence suggesting that frailty is linked to an increased risk of adverse outcomes, such as prolonged hospitalization, delirium, falls, disability, adverse drug reactions, and mortality [15, 16].

Research has suggested that frailty is a result of the combined effects of aging and certain chronic diseases [17]. A growing body of research indicates that chronic diseases, such as hypertension [18], diabetes [19], and chronic kidney disease [20], are risk factors for frailty in elderly people. Among patients with end-stage renal disease, maintenance hemodialysis (MHD) is the most frequently utilized renal replacement therapy [21]. Studies have shown that MHD patients older than 60 years account for more than 70% of all MHD patients [22]. Furthermore, research has indicated that the prevalence of frailty among elderly patients undergoing MHD is 71%, which is more than 5 times that of normal elderly people [23]. MHD and frailty may have common pathophysiological mechanisms [23], such as MHD treatment, derangements of endocrine functions, anemia, and skeletal muscle atrophy. There is growing evidence suggesting a link between MHD and frailty in older patients [24]. However, the reasons for the coexistence of MHD and frailty are not yet fully understood. Factors such as dialysis method, dialysis start time, vascular pathway types, suboptimal dialysis adequacy, metabolic disorders, increased inflammation, oxidative stress, clinical infections, anemia, and decreased activity level can contribute to a decrease in musculoskeletal mass and muscle weakness, potentially increasing the likelihood of frailty [25].

Frailty has been shown to have a negative impact on clinical outcomes in older MHD patients. A recent study confirmed that frailty is an independent risk factor for all-cause mortality in elderly patients undergoing MHD, with the mortality rate increasing by 2.83 times in those with frailty [26]. Additionally, compared with nonfrail patients, frail elderly patients on MHD experience a 1.43-fold increase in hospitalization [27]. It has also been found that frailty is a significant predictor of hospitalization in older MHD patients [27]. Previous research has demonstrated that frailty significantly increases the risk of death and falls in older individuals undergoing MHD [28]. Prevention or improvement of frailty may reduce mortality and disability associated with MHD. Therefore, recognition of frailty may facilitate the implementation of interventions aimed at preventing or delaying frailty, thereby reducing adverse clinical outcomes in older patients receiving MHD. Frailty is a controllable and potentially reversible condition [29]. Previous research has demonstrated that moderate physical exercise, appropriate dietary management, and health education can improve the overall health of frail elderly MHD patients [30].

The diagnosis of frailty in older patients with chronic diseases can provide important clinical prognostic information [31]. Assessing the frailty status of elderly patients undergoing MHD can aid healthcare providers in effectively managing disease burdens and enhancing healthcare programs and services, leading to a reduction in adverse health outcomes. Several individual studies have examined the prevalence of frailty in older MHD patients; however, there is significant variation in the findings [23, 32]. The current literature lacks consistent findings on the prevalence of frailty in older MHD patients, and no meta-analysis has been conducted. A clear understanding of the prevalence of frailty among elderly individuals undergoing MHD is needed to implement early screening and appropriate intervention strategies. This approach will help reduce suffering and mitigate negative outcomes. To address this gap, we conducted a systematic review and meta-analysis to summarize the available global epidemiological data on the prevalence of frailty and prefrailty in patients aged 60 years or older receiving MHD.

## Methods

This systematic review and meta-analysis followed the Preferred Reporting Items for Systematic Reviews and Meta-analyses [33] (PRISMA) and Meta-analysis of Observational Studies in Epidemiology [34] (MOOSE) reporting guidelines.

### Search strategy and selection of studies

In this systematic review, two researchers conducted the search, article screening, and study selection. We searched the PubMed, Web of Science, Embase, CNKI, WanFang, CBM, and VIP databases for relevant articles published from the beginning to 18 February 2023, with language restrictions. The search utilized a combination of subject words and free words and was adjusted based on the characteristics of the different databases. The search terms used were hemodialysis, maintain* hemodialysis, frailty and frail∗, and the search strategy used in the database is shown (eTable 1 in the Supplement). Initially, articles were screened based on the title and abstract, and then a second-pass screening was performed using the full text of the articles. Any disagreements were resolved by a third author (GYR).

## Study eligibility criteria

The inclusion criteria for participants were as follows: (1) had a cross-sectional or cohort design; (2) had an age ≥ 60 years or older, therapeutic hemodialysis was performed three times a week for 4 h each, and the hemodialysis period was more than 3 months. (3) Frailty was defined by frailty phenotype, frailty indices, or other forms of frailty assessment. (4) This study has sufficient data to calculate the prevalence of frailty among MHD patients. The exclusion criteria for patients were as follows: (1) studies focusing on non-English or non-Chinese articles; (2) duplicate publications, conference abstracts, letters, comments, editorials, and case reports; (3) no relevant data reported; and (4) no diagnostic criteria for frailty in MHD patients. Study eligibility was independently determined by two authors (LJJ and XWY), with any disagreements resolved through consensus with a third investigator (GYR).

## Study quality assessment and data extraction

Two authors (LJJ and XWY) assessed the methodological quality of each included study using The Joanna Briggs Institute’s Critical. Appraisal Checklist for cross-sectional studies [35] and the Newcastle–Ottawa Scale (NOS) for cohort studies [36]. The JBI checklist consists of 9 standards, and studies were considered eligible if more than 5 of the criteria were met. The NOS included 8 items assessing the selection (4 items, 4 points), comparability (1 item, 2 points), and outcomes (3 items, 3 points) of every included study, for a total score of 9 points. A score of 7–9 points represented high-quality research. Two authors independently extracted the following information from the included studies: study details (study name, authors, year of publication, country), characteristics of the research subjects (age, sample size, and proportion of women), data collection tool, prevalence of frailty and prefrailty, and research type.

### Statistical analysis

In this study, all analyses were performed using statistical software (Stata, version 16.0/IC; StataCorp LLC). An overall meta-analysis was conducted by pooling data from eligible studies using random effects models [37]. All confidence intervals are presented as 95% confidence intervals (95% CI). Higgins I-squared statistics were used to assess the heterogeneity across the included studies [38]. *I²* values of 25%, 50%, and 75% were considered low, moderate, and high degrees of heterogeneity, respectively [39].

The study assessed possible sources of heterogeneity across the included studies by considering the sex of the participants, their age, the area of study, the research type, and the assessment tools used. Meta-regression analysis was performed to explore the potential sources of heterogeneity using the abovementioned features as covariates. The relationship between study-level factors and effect size was examined through meta-regression [40]. The regression coefficient provides information on how the outcome variable changes when the explanatory variable increases by one unit [41]. The statistical significance of the regression coefficient was assessed to determine whether there was a linear relationship between the effect size and the explanatory variables. To evaluate publication bias, a funnel plot and Egger regression test were used, and the stability of the results was assessed through sensitivity analysis. A two-tailed P value less than 0.05 was considered to indicate statistical significance.

## Results

### Search results

**Fig. 1:**
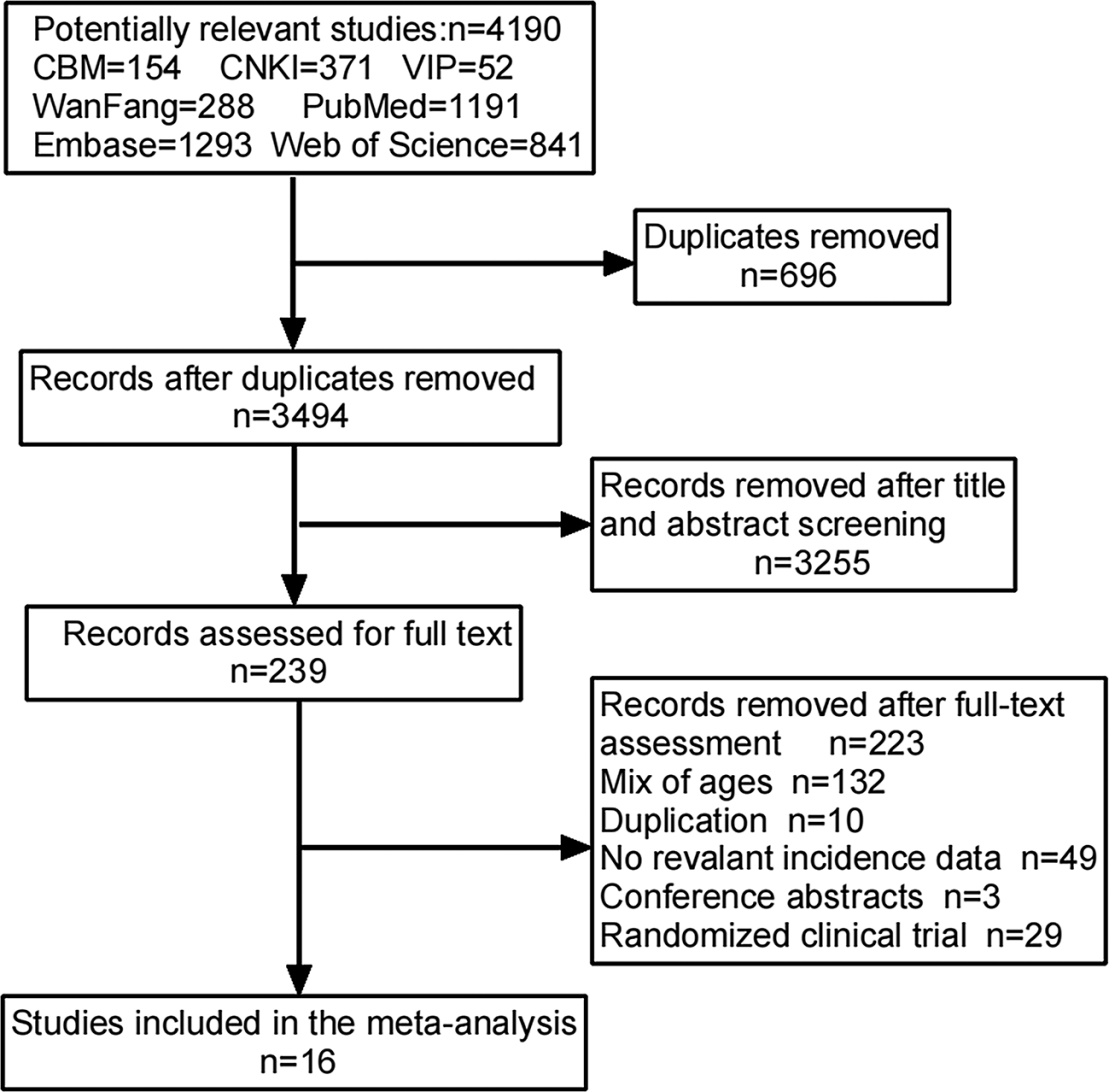
PRISMA Diagram of the study selection process

A total of 4190 records were identified from the electronic database searches for screening titles and abstracts. After removing duplicates, 3494 records were screened based on titles and abstracts, resulting in the exclusion of 3255 records. A total of 239 papers were assessed for eligibility through a full-text review. Finally, 16 studies were included in the meta-analysis. The PRISMA flow diagram was used to visualize the study selection process (Fig. 1).

## Study characteristics

The key characteristics of the 16 included studies are summarized in Table 1. A total of 2446 elderly patients undergoing MHD in 8 different countries were included in the analysis. Among these, 12 studies (75%) were conducted in Asia, 2 (12.5%) in Europe, and 2 (12.5%) in South America. The median sample size across the meta-analyses was 116, ranging from 36 to 388 participants. Of the total participants, 1072 were women (43.8%). The average age of the subjects was 70.3 years, with a range of 65 to 79.1 years. Frailty assessment varied across the studies, with 9 studies (56.1%) using either the original or modified versions of the Fried criteria, 2 studies (12.5%) using the Edmonton Frail Scale (EFS), 2 studies (12.5%) using the Tilburg Frailty Index (TFI), 1 study (6.3%) using the Fraity Index (FI), 1 study (6.3%) using the Fatigue, Resistance, Ambulation, Illness, and Loss of Weight Index (FRAIL), and 1 study (6.3%) using the comprehensive geriatric assessment (CGA). Among the 16 studies, 6 (37.5%) were cohort studies and 10 (62.5%) were cross-sectional studies. Further details regarding the characteristics of the included studies can be found in Table 1.

**Table 1:** The characteristics of the studies included in the systematic review and meta-analysis

Study name	Year	Country	Sample size	Data collection tool	Research type	Proportion of women (*n*/%)	Age	cases (*n*), Prevalence	cases (*n*), Prevalence of prefrality stage
Anna et al. [42]	2022	Italy	105	FI	②	37 (35.2)	79.1 (7.6)	58 (55.2)	-
Hidemi et al. [43]	2020	Japan	388	Fried	①	194 (50.0)	65	240(61.9)	83 (21.4)
Jyotish et al. [44]	2020	India	39	Fried	①	8 (20.5)	78.03 ± 3.90	22 (56.4)	-
Shulin Wu [45]	2021	China	264	Fried	①	106(40.2)	68.61 ± 7.59	95(35.9)	125 (47.4)
Aurora et al. [46]	2020	Spain	117	Fried	②	43 (36.8)	78.1 (4.1)	63 (53.8)	-
Zauresh et al. [47]	2020	Kazakhstan	65	EFS	①	35 (53.8)	69	15(23.1)	22 (33.8)
Yidan Guo et al. [48]	2022	China	204	Fried	②	91 (44.6)	71.65 ± 5.89	147(72.1)	42 (20.1)
Sung Woo Lee [49]	2017	Korea	46	CGA	②	17(37.0)	71.5	15(32.6)	-
Yuting Zhou et al. [50]	2021	China	315	TFI	①	141 (44.8)	66.5 ± 3.37	132(41.9)	-
Yujuan Wu [51]	2020	China	183	TFI	①	71 (38.8)	66	76(41.5)	-
Yuanyuan Li [52]	2021	China	150	Fried	②	88 (58.7)	70.25	52(34.7)	-
Yan Chen [53]	2021	China	115	Fried	②	51 (44.3)	72.1 ± 8.0	39 (33.9)	36 (31.3)
Yajie Zhu [54]	2022	China	146	FRAIL	①	55 (33.7)	67.45 ± 8.39	27(18.5)	86 (58.9)
Kai Wang [55]	2020	China	92	Fried	①	61 (66.3)	69.44 ± 8.38	31(33.7)	29 (31.5)
Juliana [56]	2015	Brazil	157	Fried	①	56 (35.7)	71.7 ± 7.7	48(30.6)	97 (6)
Fabiana [57]	2014	Brazil	60	EFS	①	18 (30.0)	71.1 (± 6.8)	23(38.5)	16 (26.7)

① Cross-sectional study; ② cohort study

FI: Fraity Index; FRAIL: Fatigue, Resistance, Ambulation, Illness and Loss of weight Index; EFS: Edmonton Frail Scale; TFI: Tilburg Frailty; Index; CGA: comprehensive geriatric assessment

## Assessment of quality of the included studies

The quality of the included cross-sectional studies was assessed using the Joanna Briggs Institute (JBI) appraisal checklist, while the quality assessment of the cohort study was conducted using the Newcastle–Ottawa Assessment Scale. The studies were found to be of moderate quality, with scores ranging from 6 to 9. The results of the quality assessment can be found in eTable 2 and eTable 3 in the Supplement.

### Prevalence of frailty and prefrailty in elderly patients with MHD

**Fig. 2:**
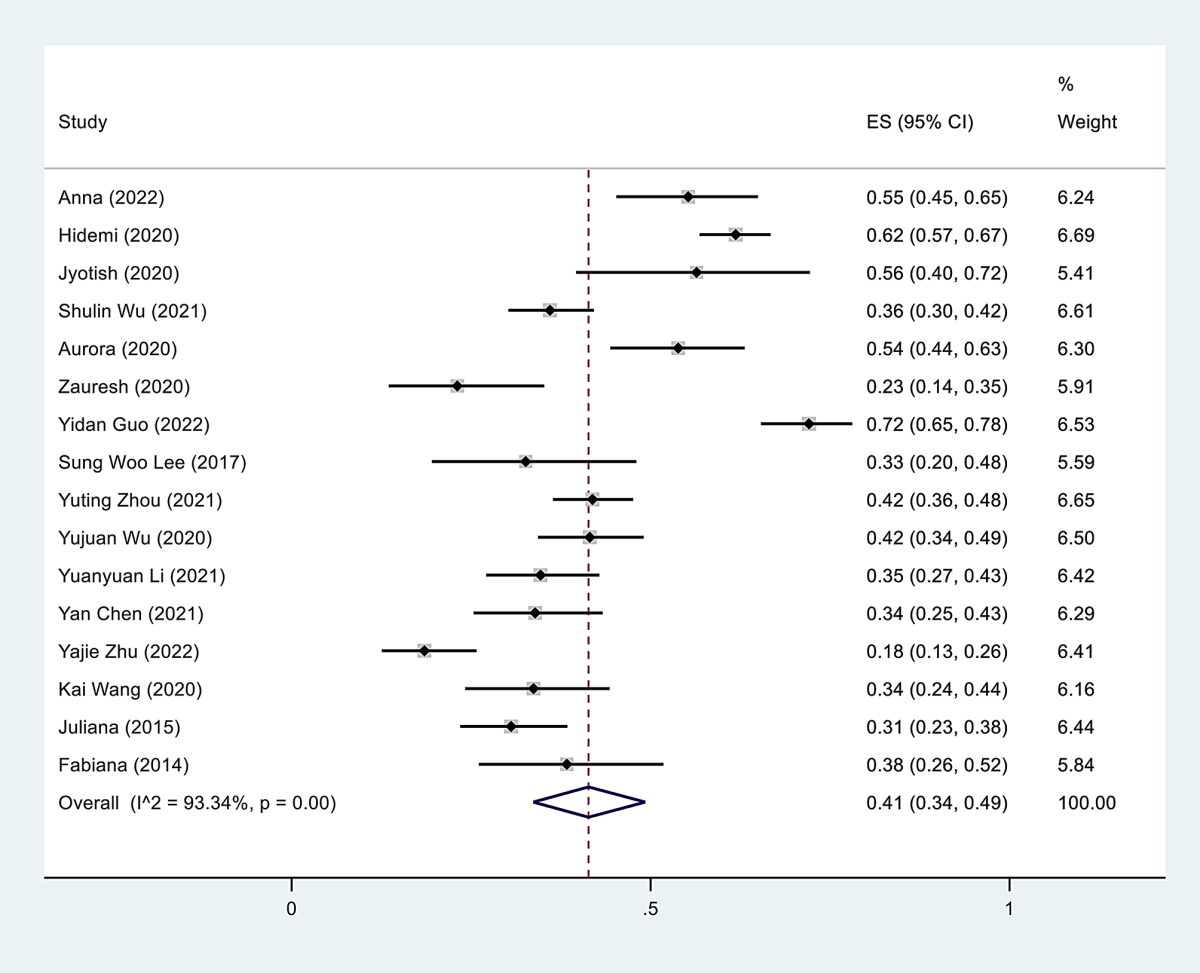
Forest plot of the prevalence rates of frailty in elderly patients on MHD

This study aimed to estimate the global prevalence of frailty in older MHD patients by analyzing sixteen studies [42–57] with a total of 2446 participants. The prevalence of frailty across these studies ranged from 18.5 to 72.1%. The pooled estimated prevalence of frailty among elderly patients with MHD was 41% (95% CI, 34–49). Notably, there was significant heterogeneity among the included studies (*I²* = 93.3%; *P* < 0.001), which is commonly observed in meta-analyses of this size. To account for this, a random-effects analysis was performed (Fig. 2). In 9 studies [43, 45, 47, 48], [53–57], the prevalence rate of prefrailty in elderly patients undergoing MHD was reported. The combined prevalence of prefrailty among 824 men and 667 women was 37% (95% CI, 26–48), with a high level of heterogeneity among the studies (*I²* = 94.9%; *P* < 0.001) (eFig. 1 in the Supplement).

### Subgroup analysis and meta-regression

The authors found a high level of heterogeneity in the effect sizes (ESs), with an I² statistic heterogeneity index of 93.3% (*p* < 0.001). To explore the potential sources of heterogeneity among the studies, subgroup analysis was conducted (Table 2). The studies were grouped based on different assessment tools (comprehensive screening tool and physical screening tool), geographical area (Asia and other areas), age (age > 70 and age ≤ 70), research type (cross-sectional and cohort study), and sex (female < 50% and female ≥ 50%). The pooled prevalence estimates for each category were similar within the subgroup analyses. However, despite these subgroup analyses, the heterogeneity between studies in different subgroups was not significantly reduced.

In the subgroup analyses, statistically significant results were observed for several factors, including assessment tools, area, age, assessment tools, and sex. It is important to emphasize that although these findings suggest a trend, the considerable overlap in confidence intervals indicates that the differences between these subgroups may not be statistically significant and may lack practical significance. For example, the prevalence of frailty identified through comprehensive screening tools (39%, 95% CI = 32 − 43%) significantly overlaps with that determined by physical screening tools (43%, 95% CI = 32 − 56%). Similarly, the prevalence in Asia (40%, 95% CI = 31 − 50%) shows substantial overlap with that in other regions (44%, 95% CI = 32 − 58%).

A meta-regression analysis was conducted to determine the study-level factors associated with frailty prevalence among elderly patients undergoing MHD. These factors included assessment tools, geographic area, age, research type, and sex. According to the multivariate random effects meta-regression (eTable 4 in the Supplement), research type was more strongly associated with a higher prevalence than was other study-level factors (**β =** -0.12, 95% CI = 0.46 − 0.23). However, the meta-regression analysis did not reveal any factors that significantly contributed to the heterogeneity.

**Table 2:** Subgroup analyses of the prevalence of frailty in older MHD patients

	Number of studies	Sample size	Heterogeneity		Prevalence	95%CI
			*P*	I²		
Assessment tools						
Comprehensive screening tool	6	774	< 0.001	74.4%	0.39	0.32–0.43
Physical screening tool	10	1672	< 0.001	95.5%	0.43	0.32–0.56
Area						
Asia	12	2007	< 0.001	94.6%	0.40	0.31–0.50
Other area	4	439	< 0.001	86.7%	0.44	0.32–0.58
Age						
Age > 70	9	993	< 0.001	92.1%	0.45	0.34–0.55
Age ≤ 70	7	1453	< 0.001	94.9%	0.37	0.26–0.48
Research type						
Cross-sectional	10	1709	< 0.001	93.1%	0.38	0.29–0.46
Cohort	6	737	< 0.001	93.4%	0.47	0.33–0.62
Sex						
Female < 50%	12	1752	< 0.001	94.0%	0.42	0.33–0.52
Female ≥ 50%	4	694	< 0.001	96.0%	0.39	0.19–0.58

### Sensitivity analysis and publication bias

To assess the reliability of the findings, a sensitivity analysis was conducted. The results of the sensitivity analysis indicated that the exclusion of individual studies did not have a significant impact on the overall measurement of the pooled effect (eFig. 2 in the Supplement). Furthermore, studies [48, 54] with a moderate risk of bias were also excluded from the analysis. Interestingly, the pooled prevalence of frailty in the low risk of bias subgroup (41%, 95% CI = 35-47%) was similar to the overall estimate (eFig. 3 in the Supplement).

In this systematic review and meta-analysis, no evidence of publication bias was found for the prevalence of frailty among elderly patients receiving MHD. This was determined through funnel plot visualization (symmetric) (eFig. 4 in the Supplement) and the Egger test (*P* = 0.213, not significant at the 0.05 level).

## Discussion

This study specifically investigates the prevalence of frailty and pre-frailty among older patients undergoing maintenance hemodialysis (MHD) on a global scale. Our aim is to provide insights into the occurrence of these conditions within this specific patient population. These findings indicate a high prevalence of frailty and prefrailty in older adults undergoing MHD. However, it is important to note that there is limited research on the long-term relationship between frailty and MHD, as only a few studies have reported such data.

The prevalence of frailty among older patients undergoing maintenance hemodialysis (MHD) is significantly higher, at 41%, compared to the 10.7% prevalence of frailty observed in community-dwelling older adults, as reported in previous studies [14]. Furthermore, the literature indicates that the risk of frailty in older adults receiving MHD is two to six times greater than that in older adults not receiving MHD [58]. The increased prevalence of frailty among older patients undergoing MHD may be associated with the detrimental effects of MHD treatment [59]. Specifically, MHD contributes to elevated levels of inflammatory cytokines, the accumulation of uremic toxins, and hyperphosphatemia, all of which can accelerate the loss of muscle mass and strength in older adults, potentially influencing the development of frailty [60]. Furthermore, hemodialysis-related anemia, neuromuscular diseases, and cardiovascular diseases such as cardiomyopathy can also impair muscle function in patients [61]. Additionally, the intake of protein and other nutrients plays a crucial role in muscle function. Research has shown that elderly patients undergoing MHD are at a higher risk of malnutrition due to protein loss during dialysis, loss of appetite, and dietary restrictions, and this malnutrition is associated with reduced body muscle strength and physical frailty [62].

High heterogeneity was observed among the included studies. To investigate the sources of heterogeneity, subgroup and meta-regression analyses were conducted to assess the characteristics of the included studies. Subgroup analysis results indicated that the prevalence of frailty was statistically significant across various frailty assessment tools, areas, ages, study types, and sex. However, the notable overlap in the 95% confidence intervals among subgroups indicates that the differences between these subgroups may lack both practical and statistical significance. Specifically, older MHD patients exhibited a higher prevalence of frailty when assessed using a physical frailty screening tool compared to a comprehensive screening tool. However, the confidence intervals for the measurements obtained from the two tools significantly overlapped, suggesting that this difference may lack actual clinical significance. The study also identified a slightly higher prevalence of frailty in individuals from regions outside of Asia. Nevertheless, due to limited sample sizes and overlapping confidence intervals, the actual significance of this regional difference remains uncertain. Additionally, a marginally higher prevalence of frailty was observed in cohort studies compared to cross-sectional studies, which aligns with the findings of previous research [25]. However, the overlap in confidence intervals suggests that this difference may not reach statistical significance. Moreover, subgroup analyses indicate that men are slightly more likely than women to experience frailty. Nonetheless, given the over-representation of males in the sample among individuals over 70 years of age, this gender difference may be attributed to sample composition rather than genuine gender effects. In terms of age, our research aligns with previous studies, indicating that the likelihood of developing frailty increases with age [63]. Nevertheless, due to the overlapping confidence intervals for age-related frailty prevalence, the significance of this trend may be diminished. Furthermore, the limited data on dialysis access type and dialysis vintage prevented us from conducting detailed subgroup analyses on these variables in our meta-analysis. Although these factors may significantly influence the prevalence of frailty, the insufficiency of data in the current study hindered relevant subgroup analyses. We recommend that future studies incorporate these variables to enhance the understanding of their impact. Therefore, clinicians and researchers should exercise caution when interpreting these findings and avoid overinterpreting trends observed between subgroups. Future studies should employ more rigorous statistical methodologies to address the challenges posed by overlapping confidence intervals and potential heterogeneity, while also incorporating additional variables such as dialysis access type and dialysis duration to achieve a more comprehensive understanding of the true contributors to frailty prevalence.

Our study has several limitations. First, we included only papers written in Chinese or English, which may have limited the scope of our review due to a lack of resources. Additionally, three-quarters of the studies included in this review were from Asian countries, potentially resulting in an underrepresentation of other regions. However, it is worth noting that most studies included in our review were from countries where English was not the native language. Second, significant heterogeneity was identified in our meta-analyses. Heterogeneity in meta-analyses of observational studies is often unavoidable and does not necessarily invalidate the results [64]. To address this, we collected prevalence data from studies that met the inclusion criteria and explored potential sources of heterogeneity using subgroup effects and random effects meta-regression. Unfortunately, we were unable to identify the specific source of heterogeneity. However, the sensitivity analysis showed the robustness of our findings. Last, it is possible that in our abstract selection process, we may have overlooked relevant studies that did not specifically focus on frailty but included information on its prevalence as a covariate.

### Research implications and future directions

Our findings may have important clinical implications. It is increasingly apparent that frailty must be considered when administering MHD treatments to older adults. Studies have confirmed that frailty is an independent risk factor for all-cause mortality in elderly MHD patients, with MHD patients who are frail having a 2.83-fold increase in all-cause mortality [26]. Our findings emphasize the significance of frailty assessment in elderly MHD patients. Frailty measurement should be integrated into clinical practice as part of routine care for older patients on MHD [6]. This approach will aid in assessing patients’ health status, predicting the risk of complications, and developing prevention strategies for frailty. Future research should focus on identifying protective factors against frailty in older adults undergoing MHD, as not all of them become frail. However, it is important to note that the availability of vulnerability data is uneven across regions, limiting our ability to accurately predict the future burden of frailty in elderly hemodialysis patients. This poses challenges in planning and allocating resources effectively for the growing population of older MHD patients [65]. Furthermore, we acknowledge that the current emphasis on maintenance hemodialysis (HD) patients may present a somewhat limited perspective. Consequently, we plan to include all maintenance dialysis patients, encompassing both HD and peritoneal dialysis (PD), in our future studies. This will enable us to conduct subgroup comparisons of frailty prevalence, thereby enhancing our understanding of the differences and similarities in frailty profiles across these two dialysis modalities.

To date, various interventions have been proven to be effective at delaying or reversing frailty in elderly MHD patients. These interventions include exercise, medication, nutrition, cognitive training, hormone therapy, and rehabilitation [66, 67]. Most interventions have shown feasibility, with compliance rates of approximately 70% [68]. However, a recent systematic review concluded that resistance training and protein supplementation have high relative efficacy and acceptability for delaying or reversing frailty among primary care interventions [69]. On the other hand, moderate-intensity exercise combined with education and health promotion within the middle range of relative effectiveness, while brief and comprehensive geriatric assessments and home visits also show midrange effectiveness. In terms of efficiency and ease of implementation, behavior change interventions were rated as low in relative effectiveness and moderate in ease of use [69]. It is important to note that most interventions are tested on frail and prefrail groups [69]. Our meta-analysis revealed that the prevalence rates of frailty and prefrailty in elderly MHD patients were 41% and 37%, respectively. This highlights the importance of tailoring intervention measures based on the disease status and physical condition of elderly patients undergoing MHD to prevent frailty and prefrailty.

## Conclusion

Our study showed that older adults receiving MHD face a greater risk of frailty, surpassing the 10.7% prevalence of frailty reported in community-dwelling older adults in previous study [14]. However, it is important to recognize that our findings may differ from the existing literature due to variations in study populations, methodologies, and other influencing factors, including differences in assessment tools, geographical regions, etc. It is crucial to enhance our understanding of the factors that contribute to this increased risk to develop effective interventions aimed at preventing or mitigating the negative effects of frailty on health.

## Electronic supplementary material

Below is the link to the electronic supplementary material.

Supplementary Material 1

Supplementary Material 2

## Data Availability

Data is provided within the manuscript or supplementary information files.
