# Supplementary material for: The prevalence of frailty among older adults with maintenance hemodialysis: a systematic
Source: BMC Nephrol. 2025 Jan 7;26:10. doi: 10.1186/s12882-024-03921-3 (PMC11724589; doi:10.1186/s12882-024-03921-3)
Supplement: Supplementary file 1 — Supplementary Material 1 [file 12882_2024_3921_MOESM1_ESM.docx]

Appendix

eTable 1 Search strategy

| **Database** | **Search strategy** |
| --- | --- |
| PubMed | **#1 (“hemodialysis”[Mesh])**  **#2 ((((((hemodialysis[Title/Abstract]) OR (haemodialysis[Title/Abstract])) OR (hemodiafiltration[Title/Abstract])) OR (renal dialysis[Title/Abstract])) OR (hemoperfusion[Title/Abstract])) OR (maintain* hemodialysis[Title/Abstract])))**  **#3 #1 OR #2**  **#4 (“Frailty”[Mesh])**  **#5 (((((((((frailty syndrome[Title/Abstract]) OR (frailty*[Title/Abstract])) OR (asthenia[Title/Abstract])) OR (frail elderly[Title/Abstract])) OR (fragile[Title/Abstract]) ) OR (hypoesthesia[Title/Abstract])) OR (Frail*[Title/Abstract])) OR (weakness[Title/Abstract])) OR (debility*[Title/Abstract])))**  **#6 #4 OR #5**  **#7 #3 AND #6**  **#8 (“renal replacement therapy” OR “hemodialysis” OR “haemodialysis” OR “hemodiafiltration” OR “renal dialysis” OR “hemoperfusion” OR “maintain* hemodialysis” ) [Text Word])**  **#9 (“Frailty” OR “frailty syndrome” OR “frailty*” OR “asthenia” OR “frail elderly” OR “fragile” OR “hypoesthesia” OR “Frail*” OR “weakness” OR “debility*”) [Text Word])**  **#10 #8 AND #9**  **#11 #7 OR #10** |

eTable 2 Qualities of the cross-sectional studies included in the systematic review and meta-analysis

| **Study name** | response | | | | | | | | | |
| --- | --- | --- | --- | --- | --- | --- | --- | --- | --- | --- |
|  | Q1 | Q2 | Q3 | Q4 | Q5 | Q6 | Q7 | Q8 | Q9 | Total |
| Hidemi et al. | Y | Y | Y | Y | Y | Y | Y | Y | U | 8 |
| Jyotish et al. | Y | Y | N | Y | Y | Y | Y | Y | U | 7 |
| Shulin Wu | Y | N | Y | Y | Y | Y | Y | Y | Y | 8 |
| Zauresh et al. | Y | Y | Y | Y | Y | Y | Y | Y | Y | 9 |
| Yuting Zhou et al. | Y | N | Y | Y | Y | Y | Y | Y | Y | 8 |
| Yujuan Wu | N | N | Y | Y | Y | Y | Y | Y | Y | 7 |
| Yajie Zhu | N | Y | Y | Y | Y | Y | Y | Y | Y | 8 |
| Kai Wang | N | Y | N | Y | Y | Y | Y | Y | Y | 8 |
| Juliana | N | Y | N | Y | Y | Y | Y | Y | U | 6 |
| Fabiana | Y | N | N | Y | Y | Y | Y | Y | U | 7 |
| Keys: | | | | | | | | | | |
| Q1–Q9 represents questions used to assess the quality of included studies, which are listed below | | | | | | | | | | |
| Q1. Was the sample frame appropriate to address the target populations? | | | | | | | | | | |
| Q2. Were the study participants sampled appropriately? | | | | | | | | | | |
| Q3. Was the sample size adequate? | | | | | | | | | | |
| Q4. Were the study subjects and setting described in detail? | | | | | | | | | | |
| Q5. Was the data analysis conducted with sufcient coverage of the identifed sample? | | | | | | | | | | |
| Q6. Was a valid method used in the identifcation of conditions? | | | | | | | | | | |
| Q7. Was the condition measured in a standard, reliable way for all participants? | | | | | | | | | | |
| Q8. Was there an appropriate statistical analysis? | | | | | | | | | | |
| Q9. Was the response rate adequate, and if not, was the low response rate managed appropriately? | | | | | | | | | | |

Y yes; N no; U unclear; NA not applicable

eTable 3 Qualities of the cohort studies included in the systematic review and meta-analysis

| Study name | Selection | | | | Comparability | Outcome | | | Total score |
| --- | --- | --- | --- | --- | --- | --- | --- | --- | --- |
|  | Representativeness  of  the exposed cohort | Selection  of  the  non exposed cohort | Ascertainment  of exposure | Demonstration that outcome  of interest was  not present at start of study | Comparability of cohorts on the basis of the design or analysis | Assessment  of outcome | Was follow-up long enough for outcomes to occur | Adequacy of follow up of cohorts |  |
| Comparability  (Anna et al. | 1 | 1 | 1 | 1 | 2 | 1 | 1 | 0 | 8 |
| Aurora et al. | 1 | 1 | 1 | 1 | 2 | 1 | 1 | 1 | 9 |
| Yidan Guo et al. | 1 | 1 | 1 | 1 | 2 | 1 | 1 | 1 | 9 |
| Sung Woo Lee | 1 | 1 | 1 | 1 | 2 | 1 | 0 | 1 | 8 |
| Yuanyuan Li | 1 | 1 | 1 | 1 | 2 | 1 | 0 | 1 | 8 |
| Yan Chen | 1 | 1 | 1 | 1 | 2 | 1 | 0 | 1 | 8 |

eTable 4 Meta-regression results of the incidence of frailty in elderly patients on MHD

| Covariate | β | SE | 95% CI | P |
| --- | --- | --- | --- | --- |
| Assessment tools | 0.03 | 0.10 | -0.18 ~0.25 | 0.73 |
| Area | -0.01 | 0.14 | -0.31 ~0.29 | 0.93 |
| Age | -0.03 | 0.18 | -0.43~0.37 | 0.88 |
| Research type | -0.12 | 0.16 | -0.46 ~0.23 | 0.47 |
| Gender | -0.002 | 0.005 | -0.014~0.010 | 0.697 |
