# Supplementary material for: The prevalence of frailty among older adults with maintenance hemodialysis: a systematic
Source: BMC Nephrol. 2025 Jan 7;26:10. doi: 10.1186/s12882-024-03921-3 (PMC11724589; doi:10.1186/s12882-024-03921-3)
Supplement: Supplementary file 2 — Supplementary Material 2 [file 12882_2024_3921_MOESM2_ESM.docx]

Appendix

eFig.1 Forest plot of the incidence rates of prefrailty in elderly patients on MHD

eFig.2 Sensetive analysis

eFig.3 Forest plot of the incidence rates of frailty in elderly patients on MHD

（excluding 2 papers with moderate risk of bias）

 eFig.4 Funnel plot symmetry
